# Public Awareness on Cancer-Associated Thrombosis among the Greek Population: First Findings from the ROADMAP-CAT Awareness Study

**DOI:** 10.1055/a-1742-0465

**Published:** 2022-01-17

**Authors:** Kyriakos Souliotis, Christina Golna, Sofia Nikolaidi, Patrick V. Dreden, Georgia Vatheia, Grigoris T. Gerotziafas

**Affiliations:** 1 Department of Social and Education Policy, University of Peloponnese, Corinth, Greece; 2 Research Department, Health Policy Institute, Maroussi, Athens, Greece; 3 Policy Change Department, Innowth, Larnaca, Cyprus; 4 Sorbonne University, Institut National de la Santé et de la Recherche Médicale (INSERM), Unit 938, Research Group Cancer, Biology and Therapeutics, Centre de Recherche Saint-Antoine (CRSA), Institut Universitaire de Cancérologie, Paris, France; 5 Department of Clinical Research, Diagnostica Stago, Gennevilliers, France; 6 Thrombosis Center, Tenon-Saint Antoine, Hôpitaux Universitaires de l'Est Parisien, Assistance Publique Hôpitaux de Paris (APHP), France

**Keywords:** venous thromboembolism, deep vein thrombosis, pulmonary embolism, cancer, public awareness

## Abstract

**Background** Cancer-associated thrombosis (CAT) is the second cause of mortality after cancer itself. CAT is underestimated as a health challenge among oncologists, whereas the levels of awareness among patients and the public have not been systematically assessed and followed in the European Union countries.

**Aim** The Prospective Risk Assessment and Management of Patient with CAT (ROADMAP-CAT) Awareness study is an investigator-initiated, descriptive and nonexperimental study with a cross-sectional design and it explores CAT risk awareness among cancer patients and the general public in Greece to provide an impetus for health policy interventions and a benchmark against which impact of any future interventions may be assessed.

**Methods** A total of 1,003 participants aged above 18 years were contacted by phone after random selection from the national telephone catalogue. Participation was voluntary and completely anonymous, and a structured questionnaire was used to elicit responses. Data were analyzed using IBM SPSS version 25.

**Results** Among respondents, almost one-third (32.3%) reported CAT awareness, while only one in five (21.7%) were aware of the signs and symptoms of venous thromboembolism (VTE). Among patients with a personal history of cancer or of VTE, 47 and 58%, respectively, were aware of CAT risk. Of those aware of the association, 35.2% identified their treating physician as the main source of information. The level of awareness did not significantly differ by responders' demographics.

**Conclusion** The ROADMAP-CAT Awareness study revealed very low levels of awareness on CAT and VTE risk both among the general public and cancer patients in Greece. Awareness of the signs and symptoms of VTE was also particularly low. Treating physicians are not actively engaging in educating their patients about CAT. Public awareness of the increased risk of VTE among cancer patients is critical to prevent and diagnose the disease early. It is imperative that a structured campaign supports medical professionals to take the time to increase awareness and educate their patients on this matter if to improve morbidity and mortality of cancer patients.

## Introduction

The association between cancer and venous thromboembolism (VTE) is well-known in the medical community and in this specific case, it is described by the term “cancer-associated thrombosis (CAT).” The risk of CAT is increased four to seven fold in patients with cancer compared with those without malignancy. Despite CAT being a preventable disease, it is a leading cause of mortality among cancer patients after cancer itself. 1 2 3 A retrospective population-based study which analyzed data from 15.7 million adult persons with cancer living in the United States showed that the overall annual incidence of CAT varied between 1.80 and 0.72% in the period 2005 to 2014, with an overall average incidence of 1.16%. Nevertheless, the incidence of CAT varied according to the type of cancer. Pancreatic cancer and anorectal cancer were associated with the highest risk of CAT. Other types of cancer, such as uterine and cervical cancer, were associated with a lower risk of CAT. Patients who were on public insurance or uninsured were more likely to have CAT than patients who were on private insurance (1.24, 1.30, and 0.92%, respectively; *p* < 0.0001). 4 Noteworthy, throughout the study period, the annual incidence of CAT decreased by 60%, but overall following an insignificant nonlinear downward trend, underlying that prevention of CAT remains an unmet need.

The risk of CAT increases by approximately six fold in outpatients on anticancer treatment or with advanced stage of the disease. 3 5 6 The mortality rate of patients with CAT is two- to three-fold higher compared with those without. Furthermore, CAT occurrence leads to modifications of the anticancer treatment schedule. Patients diagnosed with CAT must receive long-term anticoagulant therapy which exposes them to a significantly higher risk of major bleeding, thus compromising the administration of anticancer treatment. 7 In addition, CAT figures among the leading causes for prolonged hospitalization and readmissions, thus causing a substantial economic burden on the health systems. 8 The direct cost of CAT in the European Union (EU) health systems is estimated to account for €1.5 to 2.2 billion each year, mostly due to hospitalizations. 9 In France, for instance, CAT is the most frequent diagnosis leading to hospital admission in patients with breast or prostate cancer. The average cost per stay for the first thrombotic event is about €3,611. 10

CAT is an underestimated problem in the community of oncologists, despite its major impact on patient survival and quality of life and health system economics. Routine assessment of risk for CAT is recommended by international and national guidelines. 11 12 13 14 15 However, according to the European Society of Medical Oncology (ESMO) “most oncologists underestimate the prevalence of CAT and its negative impact on their patients.” 16 According to the most modern concept acknowledged by the recent recommendations published by American Society of Clinical Oncology (ASCO) experts, Oncologists and members of the oncology team should educate patients regarding CAT. 17

Thus, patients are assigned an increasingly important role in the implementation of thromboprophylaxis for CAT. In addition, proper education of patients with cancer on the recognition of signs and symptoms of VTE can lead to prompt diagnosis and a more effective treatment. Nevertheless, the levels of awareness for CAT either among patients with cancer or in the public remain low. 18 19 The evaluation of the actual state of public awareness for CAT is important for the elaboration of targeted successful communication strategies.

In Greece, public awareness of the association between malignancy and VTE is assumed to be low, particularly as the topic is largely overlooked in cancer patient information and support materials. To this end, the Prospective Risk Assessment and Management of Patient with CAT (ROADMAP-CAT) Awareness study investigated the degree of actual public awareness on the risk of CAT in a single European country (Greece) and identified variables which could contribute to the success of the communication strategy.

## Materials and Methods

### Study Design and Participants

The study was an investigator-initiated, descriptive, nonexperimental with a cross-sectional design and data were collected from September 29 to October 3, 2017. Telephone numbers were selected randomly from the national telephone catalogue and stratified according to the respondents' place of residence. Regarding the sample composition, it was weighted in terms of gender and age to match the 2011 population census, so as to be representative of the general Greek population. Participants were contacted by phone, and a standardized questionnaire was provided to the respondents with the use of computer-assisted telephone interviewing (CATI). Respondents remained anonymous throughout the whole process. When the respondent agreed to participate in the survey, an interviewer read the questions and filled in the questionnaire directly on his/her computer screen. The questionnaire was developed in cooperation with clinical experts and was pilot tested on 100 respondents before the finalization. Those responses were excluded from the final sample. Questionnaires enquired after (1) awareness of the correlation between cancer and VTE, (2) awareness of the symptoms of VTE, and (3) sources of information (e.g., physicians, relatives, and the internet). Demographic data, such as age, gender, income, and region of residence, were also collected. A section on respondents' general health and access to health services over the past 6 months was also included in the questionnaire. In total, 1,374 telephone calls were made and 73% accepted to participate in the survey.

### Statistical Analysis

The number of participants included in the study was calculated using G*Power 3.1 software, employing the following input parameters: df = 1, correlation = 0.12 (as estimates in the research area of interest are unknown, an estimate indicative of small effect was used 20 21 ), power = 0.95, error probability = 0.05, and indicated that the sample size should be 903 participants. 22 Descriptive statistics are presented using absolute and relative frequencies. Awareness of the association between VTE and cancer was assessed according to several characteristics that included age, gender, level of education, and personal and family history of cancer. For the comparison of categorical data, Chi-square tests were performed. All statistical analyses were performed using IBM SPSS version 25.

## Results

Overall, 1,003 questionnaires were completed. All respondents were 18 years of age and over. There were no exclusion criteria. A small percentage of respondents in the general public were expected to have a personal history of VTE. Therefore, responders were asked if they had ever suffered from VTE (deep vein thrombosis [DVT] or pulmonary embolism [PE]) in the past, without being excluded if their response was positive.

Among 1,003 participants in the study sample, the majority were women (54.4%), 82.9% were aged over 40 years, 71.5% were married, and 43% were secondary education graduates. Also, 67.2% resided in urban areas, 60.8% considered their income as low or low to medium, and 34.7% considered their income as medium. Among participants, 5.1% had been diagnosed with cancer. Moreover, 48.6% mentioned having a relative or close acquaintance with a cancer diagnosis and 46.1% had no cancer diagnosis experience in their social environment. Demographics data of respondents are summarized in Table 1 .

**Table 1:** Demographic characteristics ( *n* = 1,003)

	*n*	%
Gender		
Men	457	45.6
Women	546	54.4
Age (y)		
18–24	35	3.5
25–39	136	13.6
40–54	332	33.1
55–64	202	20.1
65+	298	29.7
Educational status a		
Primary education	148	14.8
Secondary education	430	43.0
University degree	355	35.5
Postgraduate degree–PhD	66	6.6
Marital status b		
Single	132	13.2
Cohabitate	14	1.4
Married	715	71.5
Divorced	47	4.7
Widowed	92	9.2
Region of residence		
Attica	382	38.1
Northern Greece	257	25.6
Central Greece	269	26.8
Aegean-Crete	95	9.5
Place of residence		
Urban	674	67.2
Rural	329	32.8
Income c		
Low	409	41.5
Low to medium	190	19.3
Medium	342	34.7
Medium to high	41	4.2
High	4	0.4
History of cancer diagnosis d		
Personal	51	5.1
Family or close acquaintance	487	48.7
No	462	46.2
Personal history of VTE e		
Yes	24	2.4
No	962	97.6

Abbreviation: VTE, venous thromboembolism.

a Missing values, 0.4%.

b Missing values, 0.3%.

c Missing values, 1.7%.

d Missing values, 0.3%.

e Missing values, 1.7%.

### Awareness of the Increased Risk for Venous Thromboembolism among Cancer Patients

Of a total of 1,003 responders, only 321 (32.3%) were aware that cancer patients are more likely to suffer from VTE. The percentage was higher among the group with a history of cancer, with 47.1% aware of the increased risk. Nevertheless, 72.9% of participants were unable to recognize signs and symptoms of VTE ( Table 2 ).

**Table 2:** Awareness of VTE ( *n* = 1,003)

	*n*	%
Are you aware of the correlation between cancer and VTE		
No	679	67.7
Yes	324	32.3
Are you aware of the signs and symptoms of venous thromboembolism, that include shortness of breath, lower limb pain, swelling and redness a		
No	729	72.9
Yes	271	27.1

Abbreviation: VTE, venous thromboembolism.

a Missing values, 0.3%.

No statistically significant association was found between awareness levels and sociodemographic characteristics. However, personal history of cancer and VTE were univariately associated with higher rates of awareness. The percentage of participants reporting awareness of the association between VTE and cancer was significantly higher among those with personal history of VTE or cancer: 58.3 versus 32.2%, *p* < 0.05, and 47.1 versus 31.6%, *p* < 0.05, respectively. Results are depicted in Table 3 .

**Table 3:** Number and percentage of respondents reporting awareness of the association between cancer and VTE by respondent characteristics ( *n* = 1,003)

	Are you aware that patients with cancer have an increased risk of venous thrombosis *n* (%)		
	No( *n* = 679)	Yes ( *n* = 324)	*p* -Value a
Personal history of VTE			
No	652 (67.8)	310 (32.2)	0.007
Yes	10 (41.7)	14 (58.3)	
Personal history of cancer			
No	649 (68.4)	300 (31.6)	0.022
Yes	27 (52.9)	24 (47.1)	
Family history of cancer			
No	342 (66.7)	171 (33.3)	0.517
Yes	334 (68.6)	153 (31.4)	
Gender			
Male	321 (70.2)	136 (29.8)	0.115
Female	358 (65.6)	188 (34.4)	
Age (y)			
18–24	25 (71.4)	10 (28.6)	0.101
25–39	95 (69.9)	41 (30.1)	
40–54	226 (68.1)	106 (31.9)	
55–64	121 (59.9)	81 (40.1)	
65+	212 (71.1)	86 (28.9)	
Income			
Low to medium	407 (67.9)	192 (32.1)	0.880
Medium	231 (67.5)	111 (32.5)	
Medium to high	29 (64.4)	16 (35.6)	
Place of residence			
Urban	452 (67.1)	222 (32.9)	0.538
Rural	227 (69.0)	102 (31.0)	
Educational level			
Primary education	106 (71.6)	42 (28.4)	0.420
Secondary education	292 (67.9)	138 (32.1)	
Tertiary education	277 (65.8)	144 (34.2)	

Abbreviation: VTE, venous thromboembolism.

a Chi-square test.

### Source of Information Regarding Venous Thromboembolism

The 321 (32%) responders who were aware that cancer patients have a higher probability of VTE were asked to identify their main source of information; 35.2% mentioned their physicians as their primary source of information and 38.7% reported being informed by nonphysicians in their social circle ( Table 4 ).

**Table 4:** Primary source of information on the association between VTE and cancer ( *n* = 324)

	*n*	%
Treating or other physician	112	35.2
A friend, relative or acquaintance	123	38.7
The internet	51	16.0
Brochures	32	10.1

Abbreviation: VTE, venous thromboembolism.

Note: Missing values, 0.6%.

Only one in five (20.3% [ *n* = 43]) of respondents who did not identify their physician as their primary source of information confirmed that their treating physician had ever discussed this topic with them.

## Discussion

The aim of the present study was to investigate the awareness on CAT and VTE among the general public in Greece. The study focused on the different factors that might affect the degree of awareness and investigate the involvement of medical professionals in public information provision.

The study highlights a generally poor public awareness of CAT. Two-thirds of respondents were unaware of CAT and 72.9% ignored the signs and symptoms of VTE. These findings are consistent with results from similar studies worldwide. Lack of awareness of CAT appears not to be influenced by sociospatial factors, the age, or the educational status of the participants. Personal history of VTE or cancer was associated with higher awareness for CAT as compared with the general population. Nevertheless, levels of awareness of 52.9% among participants with personal history of cancer reveal that that education of cancer patients on CAT risk and the benefits of thromboprophylaxis is still an unmet need. Our findings are in line with the statement of the European Thrombosis and Haemostasis Alliance (ETHA) which recognizes that low awareness for VTE symptoms, risk factors, and causes of thrombosis in the general public leads to a lack of understanding and thus a lack of notice by policymakers. 9 This lack is accentuated in patients with cancer because CAT is the second cause of death after cancer itself. Increasing awareness on CAT, as well as early recognition of signs and symptoms associated with VTE, particularly in patients with cancer, is expected to lead at the increase of thromboprophylaxis use and, consequently, reduction in VTE- related deaths.

Our findings also highlighted that the role of the treating physician was almost similar to that of friend, relative, or acquaintance, as regard to information provision on CAT to patients. This finding reflects a limited intervention of the medical community in public communication about CAT and, particularly, in patients with cancer. Although a routine assessment to identify patients at high risk for CAT is recommended by international and national guidelines, according to the European Society of Medical Oncology (ESMO) “most oncologists underestimate the prevalence of CAT and its negative impact on their patients.” 12 13 16 Data from large multinational and national surveys on patients at risk of CAT and the experience from academic medical centers involved in the management of thrombosis reveal the failure or reluctance to apply guidelines on the prophylaxis of CAT. For example, only 54% of patients receiving chemotherapy are made aware of the risk of CAT. In one French cohort, only 55% of cancer patients at risk of CAT received thromboprophylaxis. 23 In the United Kingdom, only 41% of hospital trusts have a policy for managing VTE. The reported rates of implementation of the guidelines for thromboprophylaxis in hospitalized cancer patients are lower than 18%, while the corresponding rates for hospitalized noncancer patients are higher than 50%. 24

Our ROADMAP-CAT Awareness study findings strongly support the need for a targeted communication program aimed to the general public, patients with cancer and their treating physicians on the burden of CAT, risk assessment methods, and recommended strategies for thromboprophylaxis. The data presented herein describe the actual status of awareness for CAT and will serve in the evaluation of the efficacy of targeted interventions aimed at improving current picture.

## Limitations and Strengths

Our study was conducted in the general population and therefore cannot be referred at to provide insights into specific subgroups of people, such as hospitalized patients or other at-risk populations. 25 26 The findings of this study could potentially be generalized as the sample was derived from the general public with no exclusion criteria applied.

## Conclusion

In conclusion, the ROADMAP-CAT Awareness study showed a low level of awareness on CAT in the general public and in patients with cancer. Awareness of the signs and symptoms of VTE is also particularly low. Treating physicians are not actively engaged in educating their patients and the general public about CAT. This general lack of awareness combined with the apparently low prioritization of any organized awareness campaigns on the association between cancer and VTE may be seriously undermining the morbidity and mortality indicators of cancer patients, as well as contributing to the burden cancer places on patients and family. The organization of well-designed outreach, informative campaigns on CAT, targeting particularly cancer patients, general practitioners, and oncologists is an urgent need to improve morbidity and mortality of cancer patients.

## Notes

**What Is Known about This Topic?**

- Cancer-associated thrombosis (CAT) is an underestimated problem despite its substantial impact on patient survival and quality of life and health system economics.
- Oncologists and members of the oncology team should educate patients on cancer associated thrombosis.
- Patients' knowledge of and understanding of CAT risk is expected to improve guideline implementation and increase adherence to thromboprophylaxis.
- In Greece, a country with high cancer prevalence and incidence, CAT awareness and understanding among cancer patients and the general public is underresearched, despite its potential impact on patient survival. Equally, there is very limited communication on the matter from cancer patient associations and/or relevant information sources.

**What Does This Paper Add?**

- Our study confirms very low levels of awareness of CAT risks and prevention among both cancer patients and the public in Greece as elsewhere.
- Findings of our study reported in this paper are expected to raise awareness of the actual unmet need, among health system administrators, oncology scientific societies, and patient associations and to instigate a joint effort to improve indicators through education.
- Our study adds to the existing literature and highlights an area of unmet need in cancer that is underresearched, thus hopefully, prompting more research across health systems. This would facilitate cross-system comparisons and best practice sharing to achieve optimal CAT awareness and risk management.
